# Assessing the psychometric properties of persian version of Zarit Burden interview among family caregivers of patients with multiple sclerosis

**DOI:** 10.1186/s12912-023-01260-6

**Published:** 2023-04-06

**Authors:** Hajar Haghshenas, Zeinab Jokar, Ladan Zarshenas, Mahnaz Rakhshan, Maryam Poursadeghfard

**Affiliations:** 1 Student Research Committee, Shiraz University of Medical Sciences, Shiraz, Iran; 2 Community Based Psychiatric Care Research Center, School of Nursing and Midwifery, Shiraz University of Medical Sciences, Shiraz, Iran; 3 Clinical Neurology Research Center, Shiraz University of Medical Sciences, Shiraz, Iran; 4 Community Based Psychiatric Care Research Center, Shiraz University of Medical Sciences, Namazi Square, Shiraz, Iran

**Keywords:** Burden, Caregiver, Multiple sclerosis, Iranian, Reliability, Validity

## Abstract

**Background:**

Caring for patients with multiple sclerosis (MS) imposes a great burden on caregivers and affects their lives in various aspects. This study aimed to evaluate the psychometric properties of Persian version of 22-item Zarit Burden Interview (ZBI-22) among family caregivers of patients with MS.

**Methods:**

This methodological study was conducted in Fars province, southern of Iran. For this purpose, 120 family caregivers were recruited to participate in the study from January to March 2022. Zarit Burden Interview (ZBI) was translated into Persian through forward–backward method. Face and content validity were assessed. Construct validity was assessed using exploratory factor analyses (EFA), and its reliability was assessed by measuring internal consistency and testretest stability.

**Results:**

According to face validity, the impact scores of all items were more than 1.5. Content validity ratio and content validity index values of all 22 items were 0.64-1 and 0.82-1, respectively. The scalelevel CVI/Ave was 0.97. Based on the results of factor analysis, five factors with eigenvalues more than 1 were extracted, which altogether explained 62.62% of the total variance of ZBI score. Among 22 items, one item was deleted during EFA validity assessment. Factor loading values ranged from 0.40 to 0.88. The reliability of the scale was confirmed (total Cronbach’s alpha of the ZBI = 0.88). Moreover, testretest stability assessment revealed no significant difference between test and retest scores (P > 0.05). The intraclass correlation (ICC) for the ZBI and ICCs among its factors were 0.88 and 0.6–0.86, respectively.

**Conclusion:**

The Persian version of five-factor structure ZBI can be a valid and reliable scale, and it can be used to assess caregiver burden among family caregivers of patients with MS in Iran.

**Supplementary Information:**

The online version contains supplementary material available at 10.1186/s12912-023-01260-6.

## Background

Multiple sclerosis (MS) is a debilitating disorder [1]. Most people with MS are diagnosed between the ages of 20 and 50, and the incidence of this disease in women is 2–3 times higher than that of men [2]. The prevalence of MS has an increasing trend in the Middle East [3]. MS has a high prevalence rate in Iran and it is increasing over time [4]. MS leads to complications such as walking imbalance, loss of central vision, diplopia, paresthesia, fatigue, sexual dysfunction, and speech disorders in sufferers [1].

Caring for people with MS imposes a great burden on caregivers and affects their lives in various aspects, including physical and emotional health, ethics, employment and financial status, interpersonal relationships, and social life [5]. Understanding caregiving burden or caregiver burden is important for developing and implementing appropriate interventions for caregivers [6]. Therefore, investigating and understanding the amount of caregiver burden in this special group of patients can also become a basis for carrying out appropriate interventions in order to reduce the burden and increase the quality of personal, social, and occupational life of sufferers.

Several tools have been used to measure the caregiving burden in chronic diseases, including MS. However, among these tools, the 22-Item Zarit Burden Interview (ZBI-22) scale is the most widely used tool to measure the level of burden among caregivers [7]. The psychometric properties of different forms of Zarit Burden Interview (ZBI) have been evaluated in caregivers of different diseases and in several languages, and acceptable results have been obtained [8–13]. ZBI-22 is used to measure Parkinson’s disease caregiver burden in Sweden [14], dementia caregiver burden in Italy [9], cancer caregiver burden in Mexico [10], caregiver burden of patients hospitalized in general surgery clinics in Turkey [11], and intellectual disabilities caregiver burden in Greece [13], and its psychometric properties have been confirmed. It has also been used to measure caregiver burden in patients with MS in Saudi Arabia [15] and Turkey [16].

In Iran, to the best of our knowledge, only the Persian version of its short form (ZBI-12) has been validated in spouses of veterans with chronic spinal cord injury (SCI) [17], which cannot be used to measure caregiver burden in MS before adapting it to this new population. Because, in order to use a tool in a different society, it is necessary to examine its psychometric properties in the target society [18]. Several studies have mentioned that type of disease, patients’ behavior, caregivers’ age and occupation, caregiver’s family relationship with the patient, and physical and mental conditions of patients with chronic diseases and their caregivers are related to caregivers’ experiences and caregiving burden [19–21]. Rajabi-Mashhadi et al., [17] did a study on caregivers of veterans with SCI who participated in Iran-Iraq war with a specific purpose. The attitudes, behaviors, and experiences of these caregivers might be different from those caring from patients with MS. Hence, the caregiving burden may be difference between these two populations. On the other hand, in the aforementioned study, the caregivers of patients with SCI were only their spouses, that is, all of them were women. However, in our study, all family caregivers were studied, including patients’ spouses (both male and female), children, and even parents. The children, parents, and spouses’ experience and caregiving burden can be different with respect to their age and occupation.

Considering the high prevalence and growing trend of MS in Middle East and Iran [3, 4] and given that caregivers of patients with this disease experience a special and different burden due to the age of onset for MS and its progressive nature [22], the necessity and importance of examining caregiving burden in the caregivers of patients with MS is fully recognized. On the other hand, due to the non-availability of the Persian version of this widely used and suitable tool for measuring caregiving burden, the development of an indigenous, valid, and reliable tool to measure caregiving burden among caregivers of patients with MS in the Iranian population seems necessary. This study aimed to evaluate the psychometric properties of ZBI-22 in Iranian caregivers of patients with MS.

## Methods

This methodological study was conducted in a clinic affiliated to Shiraz University of Medical Sciences. The statistical population of this study included 120 family caregivers that participate in the study from January to March 2022. After obtaining permission from the authors of the original instrument, ZBI, the study was conducted in two phases, namely the translation of ZBI and analysis of ZBI psychometric properties, including face validity, content validity, construct validity, and reliability.

### Introducing the tool

ZBI was designed by Zarit et al., (1980) to examine caregiver burden. This scale includes 22 questions and measures the physical health, mental health, social activities, and financial status of the caregiver as well as the relationship between the caregiver and the patient. Internal consistency reliability was examined using Cronbach’s alpha coefficient and item total correlation, and Cronbach’s alpha coefficient higher than 0.80 was considered as an acceptable indicator of internal correlation. The construct validity of the questions was examined and confirmed using factor analysis. Each question was rated on a 5-point Likert-type scale ranging from ‘never’ (0) to ‘almost always’ (4), with a total score ranging from 0 to 88. Higher score indicated higher caregiver burden. The scores of 0–21, 22–40, 41–60, and 61–80, respectively, indicated the absence of burden or low burden, mild to moderate burden, moderate to severe burden, and severe burden [23].

Although ZBI is identified as a non-dimensional scale by its developer, some researchers have argued that caregiver burden is a multidimensional construct and that the total score may not accurately reflect the burden experienced by the caregivers [24]. Different studies have presented different factor structures, including three-factor and five-factor models, for this scale [8, 12, 25–30].

### ZBI translation

To translate ZBI from English into Persian, the forward–backward method was used. For this purpose, two bilingual translators translated ZBI into Persian first. Then, translations were compared and merged into one single translation. The approved Persian version of the tool was given to an expert in the field of Persian language and literature to edit it in terms of vocabulary and grammar. Then, another translator was invited to backtranslate the Persian version of ZBI into English. The authors and tow bilingual translators compared the original ZBI and the translated English ZBI with each other. The translation process did not just translate word for word, but the meanings, concepts, conceptual similarities, and cultural adaptations were also taken into account. Accordingly, the Persian translation of ZBI was approved.

### Evaluation of ZBI psychometric properties

The psychometric properties of ZBI were assessed through measuring face validity, content validity, construct validity, and reliability.

### Face validity

Both qualitative and quantitative approaches were used to check face validity. In the qualitative approach, the opinions of participants (caregivers and experts) were examined. For this purpose, the questionnaire was given to 10 caregivers, and they were asked to express their opinion about difficulty, appropriateness, and ambiguity of each item, and their comments were applied. In the quantitative approach, item impact was evaluated. To do so, the importance of each item was measured based on the caregivers’ viewpoints using a 5-point Likert scale (5 = completely important, 4 = somewhat important, 3 = important, 2 = slightly important, and 1 = not important). If the impact score of the item was higher than 1.5, the item was recognized as appropriate for the further analysis and it was retained [31].

### Content validity

Both qualitative and quantitative approaches were used to determine content validity. In the qualitative approach, the questionnaire was given to 17 experts and specialists (4 senior clinical nurses and 13 individuals with PhD in nursing and health education) to give their opinions regarding the observance of grammar, the use of appropriate words, and the place of items in the questionnaire. Decisions about the deletion or retention of the items were made based on experts’ and specialists’ opinions. In the quantitative approach, in order to check the content validity ratio (CVR), the experts and specialists were asked to comment on the necessity of each item on the questionnaire using a 3-point Likert scale (3 = essential, 2 = useful but not essential, and 1 = not essential). Thus, at this stage, based on Lawshe’s table and considering the criterion of the number of experts, those items that had CVR less than 0.45 were eliminated [32].

To calculate content validity index (CVI), 17 experts were asked to rate the relevance of each item, according to the subscales of the questionnaire, using a 4-point Likert scale (1 = not relevant, 2 = somewhat relevant, 3 = quite relevant, 4 = highly relevant). CVI > 0.8 was regarded acceptable [33].

### Construct validity assessment

Construct validity was assessed through exploratory factor analysis (EFA). Several sources have indicated that 3–10 [33] or 5–10 [34] subjects can be used for each item to measure psychometric properties. Therefore, in our research, we consider the middle limit, i.e. “5 people per item” which gives a total of 110 people and we finally increased the sample size to 120 people. Family caregivers of patients with MS were selected through convenience sampling. First, the necessary permits were obtained in order to be present in the research environment and to carry out the study. The research community consisted of the caregivers of patients with MS who referred to the Comprehensive MS Center (located in a clinic affiliated with the Shiraz University of Medical Sciences). Regarding the research environment, the researcher selected the study sample from the clients on all days and hours of their presence in the clinic. When patients came to the clinic, they were first asked to introduce a family member who is their primary caregiver. The goals of the study were then explained to the caregivers with reference to the research environment. From the referring caregivers, those who were willing to participate in our study and who also met the inclusion criteria were selected. The sampling process continued until the completion of the sampling volume (120 individuals) from January to March 2022.

Inclusion criteria were having no physical or mental health problems, age > 18 years, having ability to read and write in Persian, and willingness to participate in the study. Recruited caregivers were asked to fill out the Persian ZBI through a face-to-face interview. The participants who did not respond to more than five items were excluded from the study.

Factor analyzed was done using principal component analysis (PCA) with varimax rotation. Two-tailed tests were used, and p values less than 0.05 were considered statistically significant and considering eigenvalues > 1.0 and factor loading values > 0.40. The sample was considered adequate if the Kaiser-MeyerOlkin (KMO) value was more than 0.5 [35].

### Reliability assessment

To ensure that each item of ZBI was related to the topic they were researching, internal consistency was assessed. In addition, test-retest reliability was measured to investigate the stability of ZBI scores. For internal consistency assessment, the data obtained from the 30 participants to calculate Cronbach’s alpha, then the data of the 120 participants in EFA were used to calculate Cronbach’s alpha. Cronbach’s alpha > 0.7 was interpreted as acceptable internal consistency [35]. To do testretest stability, 30 participants were asked to recomplete ZBI within a 2week interval. Then, intraclass correlation coefficient (ICC) was calculated.

### Data analysis

The SPSS (version 21) was used for statistical analysis of data. Descriptive statistics measures, such as frequency, percentage, mean, and standard deviation, were used for data presentation. The normality of data was tested using Kolmogorov-Smirnov test.

## Results

### Face validity

All 10 participating caregivers approved that the ZBI items were simple, clear, and related to caregiver burden. The impact scores of all items were more than 1.5 (Table 1).

**Table 1:** The impact scores, CVR and CVI values of the Persian version of ZBI

Items	Impact score	CVR	CVI
1 Do you feel that your relative asks for more help than he/she needs?	4.05	0.76	1
2 Do you feel that you don’t have enough time for yourself due caring for your relative?	3.36	1	1
3 Do you feel stressed for managing caring for your relative and trying to meet your other responsibilities?	4.60	0.88	0.94
4 Do you feel embarrassed due to your relative’s behavior?	1.90	0.64	0.82
5 Do you feel angry when you are around your relative?	3.36	0.88	1
6 Do you feel that your relative negatively affects your relationships with other family members or friends?	4.14	1	1
7 Are you afraid of your relative future?	4.14	0.64	0.94
8 Do you feel that your relative is dependent on you?	3.96	0.64	0.88
9 Do you feel strained when you are around your relative?	3.36	0.88	1
10 Do you feel that your health has been suffered because of your involvement with your relative caring?	3.36	1	1
11 Do you feel that you don’t have as much privacy as you would like because of caring for your relative?	3.52	0.76	0.88
12 Do you feel that your social life has been suffered because you are caring for your relative?	3.52	1	1
13 Do you feel uncomfortable when you are with your friends because of your relative presence?	2.94	1	1
14 Do you feel that your relative seems to expect you to take care of him/her as if you are the only one, he/she can depend on?	4.05	0.64	1
15 Do you feel that you can’t afford caring for your relative s?	4.14	1	1
16 Do you feel that you will be unable to take care of your relative in future?	3.44	0.88	1
17 Do you feel that you have lost control of your life since the begining of your relative’s disease?	3.44	1	1
18 Do you wish you could leave this responsibility to someone else?	2.94	0.88	1
19 Do you feel uncertain about what to do about your relative?	3.60	0.88	0.88
20 Do you feel that you should do more for your relative?	4.70	1	1
21 Do you feel that you could do a better job in caring for your relative?	4.50	0.88	1
22 Overall, how burdened do you feel in caring for your relative?	4.50	1	1

CVR: Content validity ratio, CVI: Content validity index

#### Content validity

Following qualitative assessment of ZBI content validity, the experts’ opinions regarding the use of proper grammar and appropriate words and the placement of items in their proper place were taken and 6 items were modified accordingly. According to experts, all items obtained acceptable CVI and CVR. The CVR and CVI values of all 22 items of ZBI were 0.64-1 and 0.82-1, respectively. The scalelevel CVI/Ave was 0.97 (Table 1).

#### The results of construct validity assessment

**Participants’ characteristics**: In total, 120 family caregivers responded to ZBI during EFA, the participants’ characteristics are showed in Table 2.

**Table 2:** Characteristics of family caregivers (n = 120)

Variables	n (%) or Mean ± SD
Age	43.53 ± 13.10
Gender	
Male	65 (54.2)
Female	55 (45.8)
Relationship with patient	
Spouse	55 (45.8)
Children	15 (12.5)
Siblings	10 (8.4)
Parents	34 (28.3)
Other relatives	6 (5.0)
Marriage	
Single	16 (13.3)
Married	102 (85.0)
Widowed	2 (1.7)
Education	
Primary	18 (15)
High school	12 (10)
Diploma	38 (31.7)
BS	34 (28.3)
MS	16 (13.3)
PhD	2 (1.7)
Occupation	
Employed	29 (24.2)
Worker	4 (3.3)
Self-employed	60 (50.0)
Unemployed	14 (11.7)
Housewife/house husband	1 (0.8)
Retired	12 (10)
Underling diseases	
None	80 (66.7)
Diabetes mellitus	2 (1.7)
Hypertension	10 (8.3)
Depression	8 (6.7)
Other diseases	14 (11.7)
More than one disease	6 (5.0)
Place of residence	
Shiraz city	72 (60.0)
Others Fars province cities	32 (26.7)
Other’s provinces	16 (13.3)

### Exploratory factor analysis

**Fig. 1:**
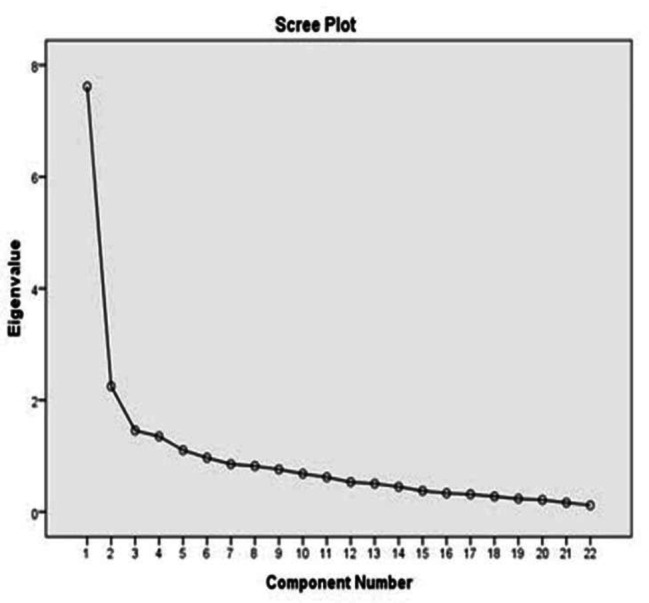
Scatter plot to determine the number of ZBI factors

Running factor analysis was appropriate as the KMO measure of sampling adequacy was 0.811 (i.e. the items measured a common factor). Bartlett test of sphericity was 1260.181 (p < 0.0001), indicating adequate sampling and suitable correlation matrix for the analysis. Based on the results of factor analysis, five factors with eigenvalues more than 1 were extracted, which altogether explained 62.62% of the total variance of ZBI score. These factors were “negative affect” (7 items: 5,9,13,16,17,18 and 22) accounting for 17.69% of the variance, “personal strain**”** (6 items: 2,4,6,10,11 and 12) accounting for 15.00% of the variance, “patient’s dependence” (3 items: 1,8, and 15) accounting for 13.19% of the variance, “uncertainly” (3 items: 3,7 and 19) accounting for 8.83% of the variance, and “guilty” (2 items: 20 and 21) accounting for 7.90% of the variance. EFA results showed that five factors were adequate for this scale (Fig. 1). Item 14 was excluded during EFA because it was not suited to any of the 5 scale dimensions. Factor loading values ranged from 0.40 to 0.88 (Table 3).

**Table 3:** Factor loading values of the ZBI items (n = 120)

Items	Factor 1 negative affect	Factor 2 personal strain	Factor 3 patient’s dependence	Factor 4 uncertainly	Factor 5 guilty
5 Do you feel angry when you are around your relative?	0.40				
9 Do you feel strained when you are around your relative?	0.54				
13 Do you feel uncomfortable about having friends over because of your relative?	0.79				
16 Do you feel that you will be unable to take care of your relative much longer?	0.82				
17 Do you feel you have lost control of your life since your relative’s illness?	0.70				
18 Do you wish you could leave the care of your relative to someone else?	0.78				
22 Overall, how burdened do you feel in caring for your relative?	0.51				
2 Do you feel that because of the time you spend with your relative that you don’t have enough time for yourself?		0.58			
4 Do you feel embarrassed over your relative’s behavior?		0.56			
6 Do you feel that your relative currently affects our relationships with other family members or friends in a negative way?		0.64			
10 Do you feel your health has suffered because of your involvement with your relative?		0.68			
11 Do you feel that you don’t have as much privacy as you would like because of your relative?		0.57			
12 Do you feel that your social life has suffered because you are caring for your relative?		0.63			
1 Do you feel that your relative asks for more help than he/she needs?			0.60		
8 Do you feel your relative is dependent on you?			0.80		
15 Do you feel that you don’t have enough money to take care of your relative in addition to the rest of your expenses?			0.59		
3 Do you feel stressed between caring for your relative and trying to meet other responsibilities for your family or work?				0.50	
7 Are you afraid what the future holds for your relative?				0.69	
19 Do you feel uncertain about what to do about your relative?				0.68	
20 Do you feel you should be doing more for your relative?					0.81
21 Do you feel you could do a better job in caring for your relative?					0.88
Variance (%)	17.69	15.00	13.19	8.83	7.90

The items selected for each of the 5 factors obtained in this study were different from those of other studies conducted in other populations and cultures. In other words, all the items in each factor were not exactly the same as the items included in similar factors in other studies, except for the last factor, that was, “guilty”.

### The results of reliability assessment

Results of the study indicated that ZBI had acceptable internal consistency (Cronbach’s alpha = 0.88). Moreover, testretest stability assessment was done by using paired sample t-test. The findings revealed no significant difference between test and retest scores (P = 0.895). Paired sample t-test showed high correlation between test and retest results. The ICC for the ZBI and ICCs among its factors were 0.88 and 0.6–0.86, respectively. These findings confirmed the internal consistency and the stability of the Persian version of ZBI.

## Discussion

This study aimed to evaluate the psychometric properties of the Iranian version of ZBI among family caregivers of patients with MS. Based on our findings, the ZBI items were comprehensible for the caregivers of patients with MS and were appropriate for the Iranian culture and context. Following quantitative and qualitative assessment of content validity, it was found that the CVR and the CVI of all 22 items were acceptable. According to EFA, the five factors of “negative affect” (7 items), “personal strain**”** (6 items), “patient’s dependence” (3 items), uncertainly” (3 items), and “guilty” (2 items) explained 62.62% of the total variance. Item 14 was excluded during EFA because it was not suited to any of the 5 dimensions. Furthermore, our findings revealed that this scale was reliable. To the best of our knowledge, our study was the first study evaluated the psychometric properties of the Iranian version of ZBI among family caregivers of patients with MS in Iran. Our study findings revealed that the Persian ZBI could be an appropriate scale for the assessment of caregiver burden among the Iranian family caregivers of patients with MS.

In this study, when doing qualitative assessment of content validity, 6 items were modified according to experts’ opinions regarding the use of proper grammar and appropriate words and the placement of items in an appropriate place. In Özer et al.‘s study, the panel of experts did not suggest any modification or change in the scale following content validity evaluation, confirming the clarity and content validity of the items [11]. Caregiving burden is a multidimensional construct [24], and the results of the present study, in line with studies done by Tang et al., [28], Lu et al., [8], and Ko et al., [27] supported the 5-factor structure of this scale. However, the items selected for each factor were different from each other across these studies. This difference may be related to the difference in the selected caregiver samples and the techniques used [29], because the levels of caregiver participation in the care of people with different diseases can be different. Furthermore, EFA is often considered a rather subjective statistical method, and different choices of data analysis methods and different criteria used to retain factors may lead to various factor models [36].

In our study, based on EFA results, “negative affect” with 7 items was identified as the first extracted factor with the highest total variance. This dimension was termed by other researchers as negative emotion [27, 28] or psychological burden [26]. After assessing item content, we found that this factor encompassed several complicated feelings of caregivers, including anger, strain, concern, uncomfortability, and annoyance; therefore, we named it negative affect.

The second factor with the highest total variance was “personal strain”, which was labelled as embarrassment/anger [25] or consequences of caregiving [12] in other studies. In this study, we named it personal strain because all of these items described the impact of caring for patients with MS on caregiver’s personal life. The third factor was “patient’s dependence”, which was also found in other studies [27, 28].

The forth factor was “uncertainly”, which was also reported as uncertainty about patient’s future in previous studies [27]. This factor showed the fear and uncertainly of caregivers about their patients care and future.

The last extracted factor was “guilty” comprising of only two items (items 20 and 21). It was labelled as self-accusation and guilt [28], self-criticism [8, 25, 30], and inadequacy [27] in other studies. Despite the fact that previous studies investigated the psychometric properties of ZBI in different populations and cultures, this factor showed the most generalizability and stability [8, 25, 27, 30]. An additional table file shows the factors and items of each factor in different studies confirming the 4- and 5-factor structure of this scale [see additional file 1].

Our study findings also revealed that the total Cronbach’s alpha of ZBI was 0.88. Moreover, testretest stability assessment revealed that there was no significant difference between test and retest scores (P > 0.05). The ICC for the ZBI and ICCs among its factors were 0.88 and 0.60–0.86, respectively. Results of a Chinese study showed that the Cronbach’s alpha of the final model was 0.88. Internal consistency coefficients of individual subscales ranged from 0.68 to 0.84 [28]. Other studies also reported that the Cronbach’s alpha of the Turkish, Chinese, Italian, English, and Mexican versions of ZBI were 0.82 [11], 0.87 [8], 0.90 [9], 0.91 [37], and 0.90 [10], respectively.

Due to the COVID-19 pandemic, we faced sampling limitation, so this study was conducted only in one academic center and relatively small sample size was included. In addition, only caregivers of outpatients participated in the present study. Hence, future studies are suggested to select participants from different centers and include caregivers of hospitalized patients using larger sample size. Also, in this study the factor analysis method for construct validity was used and criterion validity was not performed. Unfortunately, we had to exclude item number 14 because it did not match any of the factors. Finally, future studies are recommended to evaluate the criterion validity and using confirmatory factor analysis to confirm the extracted factors.

## Conclusion

This study results supported that the Persian version of ZBI, as a five-factor structure, was a valid and reliable scale. Nurses can use this scale to assess caregiving burden and needs of the caregivers of patients with MS in Iran to plan suitable interventions to reduce caregiving burden in different dimensions and improve patients’ quality of life. The use of this scale is suggested in future research and in the clinical settings.

## Electronic supplementary material

Below is the link to the electronic supplementary material.

Supplementary Material 1

## Data Availability

The datasets during the current study are not publicly available due to confidentiality of the patients’ data, but they will be available from the corresponding author on reasonable request.

## Abbreviations

MS: Multiple Sclerosis
ZBI-22: 22-Item Zarit Burden Interview
ZBI: Zarit Burden Interview
EFA: Exploratory Factor Analyses
KMO: Kaiser–MeyerOlkin
CVR: Content Validity Ratio
CVI: Content Validity Index
PCA: Principal Component Analysis
ICC: Intraclass Correlation Coefficient
SPSS: Software package used for Statistical Analysis.
