# Supplementary material for: Assessing the psychometric properties of persian version of Zarit Burden interview among family caregivers of patients with multiple sclerosis
Source: BMC Nurs. 2023 Apr 6;22:97. doi: 10.1186/s12912-023-01260-6 (PMC10077318; doi:10.1186/s12912-023-01260-6)
Supplement: Supplementary file 1 — Supplementary Material 1 [file 12912_2023_1260_MOESM1_ESM.docx]

**Additional file 1:** ZBI factors reported in different studies

| Factor name and items | Factor numbers | Study |
| --- | --- | --- |
| -Consequences of caregiving: 2,3,5,6,9,10,11,12,15 and 17  -Patient’s dependence: 1,8 and 14  -Exhaustion and uncertainty: 4,13,16,18 and 19  -Guilt and fear for patient’s future: 7,20 and 21 | 4 | Al-Rawashdeh et al (12) |
| -Caregiver’s over sacrifice: 2, 3, 10, 15, 16, 17, 18 and 22  -Patient’s dependence: 8, 11, 12 and 14  -Negative emotion: 4, 5, 6, 9 and 13  -Inadequacy: 20 and 21  -Uncertainty about patient’s future: 1, 7 and 19 | 5 | Ko et al (27) |
| -Negative emotion: 4,5,7,9,10,15,16,17,18 and 19  -Interpersonal relationship: 6,11,12 and 13  -Time demand: 1,2 and 3  -Patient’s dependence: 8 and 14  -Self-accusation and guilt: 20 and 21 | 5 | Tang et al (28) |
| -Personal strain: 1, 2, 3, 4, 6, 7, 8, 9 and 10  -Captivity: 11, 12, 13 and 14  -Loss of control: 16, 17 and 19  -Self-criticism: 20 and 21 | 4 | Cheng et al (30) |
| -Sacrifice: 3, 7, 8, 10, 11, 12, 13 and 14  -Loss of control: 15, 16, 17 and 19  -Embarrassment/anger: 4, 5, 6 and 9  -Self-criticism: 20 and 21  -Dependence: 1, 2 and 18 | 5 | Lu et al (8) |
